# DAS Writeback: A Collaborative Annotation System

**DOI:** 10.1186/1471-2105-12-143

**Published:** 2011-05-10

**Authors:** Gustavo A Salazar, Rafael C Jimenez, Alexander Garcia, Henning Hermjakob, Nicola Mulder, Edwin Blake

**Affiliations:** 1 Computer Sciences Department, University of Cape Town, South Africa; 2 Computational Biology Group, Department of Clinical Laboratory Sciences, University of Cape Town, South Africa; 3 European Bioinformatics Institute, Hinxton, Cambridgehire, UK; 4 Faculty of Languages and Literary Studies, Bremen University, Germany

## Abstract

**Background:**

Centralised resources such as GenBank and UniProt are perfect examples of the major international efforts that have been made to integrate and share biological information. However, additional data that adds value to these resources needs a simple and rapid route to public access. The Distributed Annotation System (DAS) provides an adequate environment to integrate genomic and proteomic information from multiple sources, making this information accessible to the community. DAS offers a way to distribute and access information but it does not provide domain experts with the mechanisms to participate in the curation process of the available biological entities and their annotations.

**Results:**

We designed and developed a Collaborative Annotation System for proteins called DAS Writeback. DAS writeback is a protocol extension of DAS to provide the functionalities of adding, editing and deleting annotations. We implemented this new specification as extensions of both a DAS server and a DAS client. The architecture was designed with the involvement of the DAS community and it was improved after performing usability experiments emulating a real annotation task.

**Conclusions:**

We demonstrate that DAS Writeback is effective, usable and will provide the appropriate environment for the creation and evolution of community protein annotation.

## Background

The annotation of biological data is a common task in different fields of the life sciences, and can be classified into two types: manual and automatic [1]. Manual annotation refers to the actions of an individual, usually an expert in the field, annotating the evidence extracted during a review of published scientific literature. It is a valuable effort that produces important resources like UniProtKB/Swiss-Prot, a manually annotated database of high quality protein information [2]. Automatic annotation is generally based on the hypothesis that two very similar sequences (homologues) have a common ancestor and their functions and features should be similar; therefore, any annotation in one of the sequences can be extrapolated to the other. Automatic annotation is required because of the flood of data that can not be handled manually; genome projects, among others, are able to generate terabytes of information on a daily basis and it is therefore impossible to have enough experts to annotate this quantity of data manually. However, automatic processes are inexact [3], they can infer erroneous annotations.

A combination of the two types of annotation is required in order to balance the needs for both high quality annotation and large-scale processing. Manual annotation thus becomes a quality-control mechanism for the information obtained by automatic methods. Currently, most manual annotation is performed by experts employed by the institutions hosting databases, but many additional experts in the wider scientific community could contribute to this effort if the facilities existed to do so. We have designed and implemented the Distributed Annotation System (DAS) Writeback, which enables community-based manual annotation of public data. Our approach makes the process of manual annotation a collaborative task, whereby any individual can participate by sharing their knowledge in the form of new or edited annotations.

Collaborative environments such as WikiProteins [4] or Gene Wiki [5] use the wiki paradigm in the biological domain. The central goal of WikiProteins is to promote the community annotation of biomedical concepts and their interactions, however it does not offer any tools for annotating specific biological parts of a protein -e.g. positional features. In contrast to the wiki-based approach, which duplicates information from its original source to make it part of the wiki environment, DAS Writeback directly references the source database. DAS allows the user to access several sources in a federated way and at the same time use tools for editing the data. DAS [6] is a widely-adopted standard communication protocol, and has an established set of methods to make the annotations from different network locations available in the same context. Annotations in DAS are known as features, and each has a defined set of attributes. For example: *TYPE *indicates the type of the annotation, *START-STOP *define the position, and *METHOD *describes the method used to identify the feature. DAS is motivated by the idea of providing a federated system; a logical association of independent sources distributed over multiple sites, which provides a single, integrated, coherent view of all resources in the federation. This architecture makes several distinct physical data sources appear as one logical data source to end-users. Here we describe the implementation of a DAS Writeback system through an extension of the existing DAS protocol. We present an example of the system and results of a usability experiment to test the implementation.

## Implementation

A Masters thesis at the Chalmers University of Technology on this topic resulted in the implementation of a DAS Writeback server as a proof of concept [7]. The graphical user interface was built using JSP (Java Server Pages) and the servers were Java servlets. The mechanism used to store the new annotations was incompatible with the concept of meta-annotation, which is one of the fundamental ideas of this project. Despite this, the experiences and results of that project were very useful and enabled us to avoid several potential issues.

A fundamental distinction between the previous project and the one presented here, is that the former adopts the proposals included in the DAS/2 document, whereas we propose an extension for DAS 1.6. Please note that DAS/2 is an entirely separate specification which is not backwards compatible with existing servers and clients despite being based on the DAS architecture. The submission forms used in the previous project acted as the start point to get a more specific form for protein annotation, to which we added some user interface aids that are discussed below. Furthermore, we thought that the user should be immersed in the context of the proteins that they are annotating, and therefore decided to embed the writeback functionality in a DAS client, which provides the available information for the target protein. The DAS Writeback system provides the capabilities of reading, writing, editing and deleting features by users of a web application. For the design and development of such a system it was necessary to design an architecture that supports the new features, define an extension of the DAS specification to accommodate the client-server communication, and implement server and client components. All of these milestones were achieved while trying to follow the same style as the existing DAS technology, thus looking for an easy adoption of the system by the DAS community.

When extending the DAS protocol to support servers that can store edited annotations, we set out to retain compatibility with the existing read-only system of HTTP GET requests. Development was based on the idea that a DAS Writeback server should have, at the very least, the methods for basic reading/writing operations. In Database Theory, this is known as CRUD (Create, Read, Update and Delete) [8].

The components of the system were developed bearing the following goals in mind: 1. The original annotations of a DAS source should not be modified directly; 2. The system should be trusted by the user; and 3. The system should promote interaction between the server and users.

### Architecture

In order to accomplish the first goal, the architecture includes a third party writeback server that stores the changes to a set of annotation, independent of the original source providing those annotations. In addition, changes to annotations can be considered annotations themselves and so the writeback server must provide methods to annotate annotations. This requires three new types of annotation: Create, Update and Delete.

**Figure 1:**
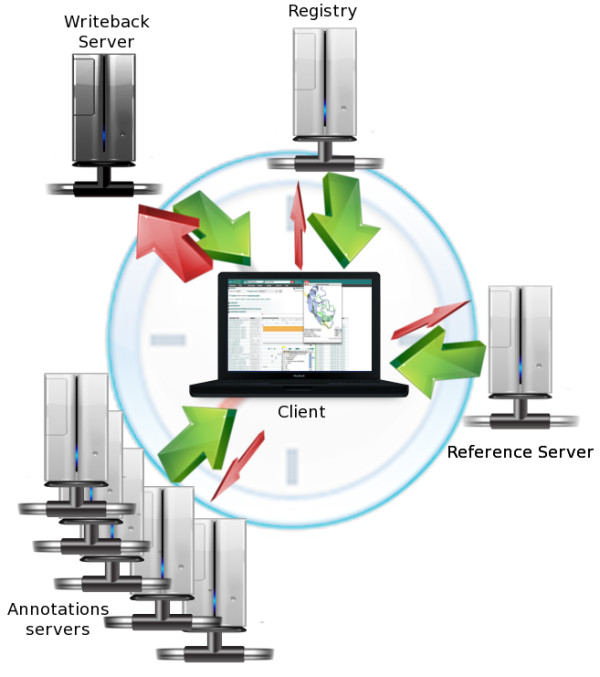
**Writeback in the DAS Architecture**. Extension of the DAS architecture for the writeback. A third party writeback server is the last to be queried by the client, and its response is used to update the information provided by the annotation servers. Communication with the writeback server has the peculiarity that the amount of information sent by the client is considerably larger than for any other server. The clock in the background represents the chronological order of the actions in a DAS transaction.

Figure 1 represents the architecture of DAS including the writeback server. Firstly it is necessary to highlight that, when a feature is requested, the writeback server behaves as another annotation server, but is the last one in the queue. The way this information is rendered is the responsibility of the client. A standard DAS transaction starts by querying the DAS Registry (the DAS Registry provides a repository for the registration and discovery of DAS services). Next the reference sequence is obtained, followed by parallel requests to several annotation sources. The interaction between client and writeback occurs after the client has retrieved and displayed all of the information for the target protein, since it is only then that the user has a complete landscape view to take the decision to add, update or delete a feature. HTTP requests relating to write operations on the writeback server are much larger than standard DAS requests (shown in Figure 1 as the width of the red arrow). The reason for this is that the client is now required to send the information to add or update a specific feature, including its type, category, position and other characteristics predefined in DAS. The communication with the writeback server is thus extended beyond the display of a graphic that compiles the information from all the servers. This is when the user starts to interact with the information, transforming the client from a pure visualisation tool to an interactive interface between the user and the DAS data.

### Protocol Extension

RESTful web services implement remote procedure calls across the Web as an alternative solution to SOAP (Simple Object Access Protocol) web services. One of the major strengths of the RESTful strategy is that it is based on such widely adopted standards as HTTP, XML, URI and MIME. This makes REST, and therefore DAS, technologies easy to implement and attractive to both developers and final users. A comparison between SOAP and REST web services can be found in [9].

One of the main features of the REST architecture is to have a uniform interface. This means that REST resources should be manipulated using a predefined set of operations. In the case of the Web, those operations are the 4 basic reading/writing operations CRUD, and the HTTP methods PUT, GET, POST and DELETE are suggested in the literature to specify those actions. These operations "*are broadly applicable but they also help uphold specific Web architectural properties" *[10].

The idea of specifying operations for publishing and editing resources using HTTP is not novel; AtomPub is a proposed protocol for publishing and editing Web Resources using HTTP [11]. Google has also defined a protocol based on Atom, AtomPub and RSS2.0 [12]. The writeback specification used for this implementation is a combination of features of those protocols, plus the inherent requirements of the DAS technology. The proposed specification can be found on the DAS1.6E web page (http://www.biodas.org/wiki/DAS1.6E#DAS_writeback 2009). It proposes that both input and output documents for the writeback should follow the DAS GFF format (See Additional File 1); the HTTP method indicates what to do with the received document (create, update or delete a feature) and the HTTP status codes used for DAS remain valid and will indicate success or failure of the requested command.

### Server

Our implementation of DAS Writeback is an extension of the MyDAS server [13] and is based on DAS1.6. A writeback data source was implemented to store annotations. Annotations are the main entity in the data model, and any edits or deletions of an annotation are considered to be versions of the original annotation.

The datasource uses Hibernate [14] as its layer to access the persistence data, which brings the advantage of being *Database-Engine *independent. The data source has been successfully tested using PostgreSQL, MySQL and Derby but is expected to work properly in other engines.

### Client

As a federated system, DAS delegates most of the integration responsibilities to its clients, giving it a *"dumb server, clever client" *architecture [15]. As a consequence, if the goal is to capture feedback from users (Writeback), the client should be able to execute several tasks related to both logic and user interaction. One of the goals of this project was to create the perception for users that the writeback functions in a client are native and can be used naturally with existing clients. For this reason, the extension of an existing client was preferable to implementing a new client from scratch. In addition, the writeback server behaves as any other DAS server for reading purposes, so many software routines of an existing client could potentially be reused for the writeback visualisation.

Dasty2 [16] is a web-based protein DAS client, which makes extensive use of AJAX in order to make the user's experience as close as possible to using a stand-alone client. Dasty2 offers a number of features that make it an ideal candidate for the proposed extensions. For example, Dasty2 has a modular structure based on panels, so it provides the opportunity to group the writeback features in a new panel, thus isolating the writeback content for those who prefer not to use this information. Dasty2 went through a refactoring process, optimising its code to provide a plug-in framework. The new version is called Dasty3. The writeback client has been implemented as a Dasty3 plugin and is included in its core feature set.

The communication between the client and the writeback server has some differences with respect to the communication with other DAS servers. Firstly, the different HTTP methods (PUT, GET, POST and DELETE) should be used according to their function. For this reason, the proxy component of Dasty3 was extended to support the appropriate method usage. The second difference is in the amount of information transferred; before the writeback, all the requests in Dasty3 were using the *GET *method. Therefore the information sent from the client to the proxy was limited to 256 characters, which is the URL size limit for some web browsers and servers. With the writeback functionalities, however, the client sends an XML document that is likely to exceed the URL size limit, making the use of other HTTP methods mandatory. This reinforces the applicability of the choice of adopting the RESTful standard. The communication between writeback client and server is achieved using the DAS GFF XML format, which is defined in the DAS specification. The client has a logical model to map the DAS GFF format when it is reading from the writeback server, and also starts from this model to build the XML when information is to be sent to the server.

## Results

We have developed a DAS writeback tool by extending existing DAS clients and servers. The writeback is included as a plug-in of Dasty3 and is integrated in the latest implementation of MyDAS, compliant with the current DAS 1.6 specification. The extensions performed in Dasty3 in order to support the writeback capabilities are divided below into reading and writing functions, i.e. if annotations are requested or if a change/creation is submitted, respectively:

### Reading Functions

**Figure 2:**
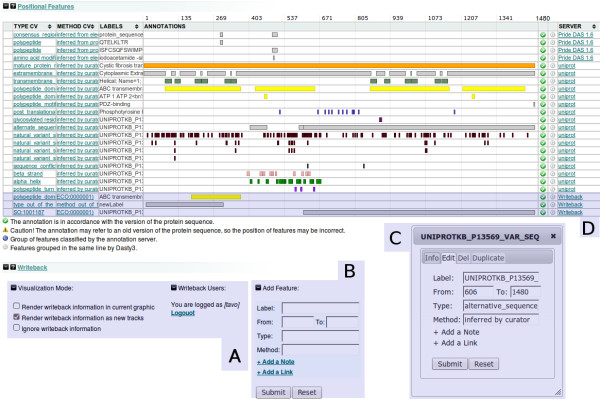
**Dasty3 + Writeback**. Snapshot of Dasty3+Writeback highlighting some of the modified/added features on dasty3 to support the writeback capabilities.

The writeback server behaves like any other DAS source when a set of features is requested. The client decides when and how to process this information. For the Dasty3 writeback plug-in, the user has three different modes to operate (Figure 2A):

#### Disable the writeback display

The first mode essentially ignores the writeback information and in this case Dasty3 just collects and displays the original information from the sources. This is useful for the users who do not want the collaborative information displayed.

#### Writeback as an extra source

Dasty3 can display the information coming from the writeback server as an extra data source. In this case, all the writeback features will be displayed as new tracks, allowing the users to compare the original annotation with the last version of it in the writeback server. Figure 2D shows an example of this display.

#### Merging the writeback with the sources

In this mode, the writeback annotations overwrite the original ones in the graphic. This generates a similar graphic for features as normally rendered by Dasty3, but incorporating the modifications that the writeback server contains. The features tagged as deleted will be transparent in the graphic, and just its border will be visible.

### Writing Functions

**Figure 3:**
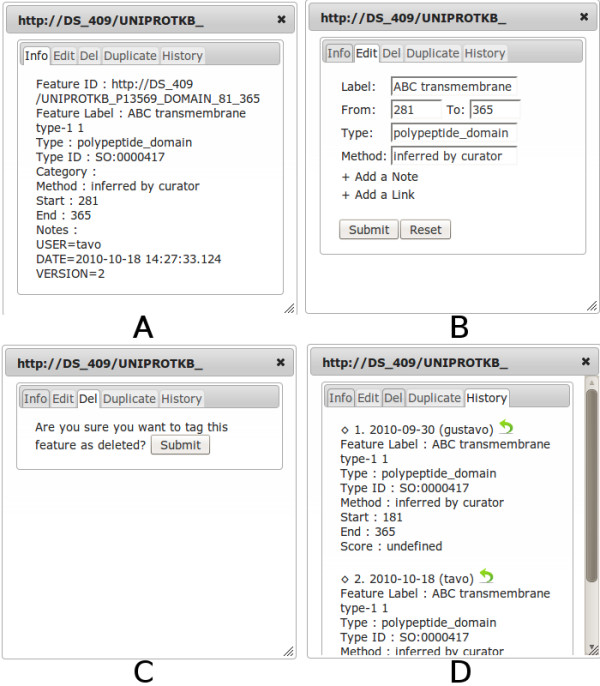
**Tabs for writeback functions in Dasty3**. From left to right: (a) Detailed information of the feature. (b) Form to edit any detail of the feature. (c) Confirmation for deletion. (d) Writeback history of the feature

After authentication, the writeback extension for Dasty3 allows users to Create, Update and Delete features. The internal pop-up windows of Dasty3 are reused in order to display the tools to execute these functions in the same context as the selected feature's information. With this goal in mind, a set of tabs was added to these windows. Figure 3 shows the contents of the four different tabs that the user can choose after clicking on a particular feature. The first tab (Figure 3A) is the detailed information that Dasty3 provides to the user for the chosen feature, the other three tabs give access to the writeback capabilities. Below is a description of how the writeback capabilities are made available in Dasty3:

#### Update

Figure 3B is a screenshot of the edit tab; in it the user has the same detailed information, but in a form that allows the user to change the values of any field. When the information is sent to the server, it is stored as the current version of the feature and it will be the one to which the server returns for future requests. Another way to edit a feature is through the history tab (Figure 3D). In this case, the user can choose to roll-back to a previous version.

#### Create

In the top-right corner of the writeback panel (Figure 2B) there is a form to add a new feature, which is similar to the one in Figure 3B but without any content in the fields. The user enters the details of the feature in the form, Dasty3 sends them to the writeback server and a new feature is created.

#### Delete

Figure 3C shows a confirmation message for the deletion of the feature. Features are not really deleted from the server, rather they are tagged in such a way that this information can be used to hide the features in the *merge *method. The list of current deleted features is displayed in the writeback panel.

### User Interface Aids

Version 1.6 http://www.biodas.org/documents/spec-1.6.html of the DAS specification recommends the use of ontologies in order to standardise both types and evidence codes, and make the task of integrating annotation from several servers easier. The recommendation says that for the values of the attribute cvId and the content of the element TYPE, the SO (Sequence Types and Features), MOD (Protein Modifications) and BS (BioSapiens Annotations) ontologies should be used. In the case of the method, the ontology to use is the Evidence Code Ontology. [15].

In order to promote the use of those ontologies, a list of suggested terms from the corresponding ontology is displayed in the edit form (Figure 3B) while the user is writing in the fields "type" and "method".

The same form has a set of logic validations to ensure that the coordinates of the annotation are not out of the limit imposed for the size of the protein, and that the start amino acid is before the end amino acid. Finally, the orientation and phase components of a DAS feature are defined by default to *Non Applicable *because these genomic-specific fields do not apply to annotations of proteins.

A basic module to allow for user authentication through a login and password was added in the writeback panel (Figure 2A). Any writing function is conditional on prior login and password validation. The reading functionality does not require authentication.

### Usability Experiment

At the conclusion of two cycles of design, implementation and feedback from the DAS community, we subjected the system to a final formative evaluation by conducting a usability experiment. The technique used to design such an experiment was Constructive Interaction [17]. Basically, Constructive Interaction consists of executing the tasks in dyads, one of the users is the actor (who has control of the computer) and the other is the co-actor. The instructions for the test subjects indicate that they consult each other before any action and avoid contact with the facilitator. In this way the ideas are expressed more naturally as a normal communication between the parts of the dyad.

The experiment was executed with the participation of eight postgraduate students organised in dyads. The annotation tasks were based on data extracted from a published paper, demonstrating that the system can be used for a real biological use case. All the sessions were recorded and analysed for further improving the writeback extension. Details of the usability experiment are described in [18].

The experiment allowed us to capture fifteen usability issues. Only one was classified as a Major Problem(In Dasty, the 'positional features' table was not automatically updated after the first added annotation) and five as a Minor Problems. There were two positive findings, two bugs and five suggestions. A detailed list of the findings is included in the Additional File 2. All the problems and bugs were solved for the final version of the application, three of the suggestions were implemented and the remaining two were postponed to a future maintenance cycle. The major outcome of the experiment is that the users were able to use the writeback functionalities without extensive training, giving us two important things to highlight: Firstly, both server and client function according to the user's expectations, and secondly, the functionalities are intuitive enough to allow untrained users to solve protein annotation tasks.

## Discussion

At the time of writing this manuscript, the DAS registry reports over 1200 data sources. This illustrates the high adoption of DAS, making it the perfect environment for a collaborative approach as presented here. The writeback specification is now an official extension in DAS and is considered to be a part of the core protocol. The developed software has been well received by the community. On the one hand, the server implementation is now part of the official development of one of the more stable DAS servers (myDas); and, on the other hand, the client is included in the set of plugins of Dasty3, which is a widely used DAS client. However, the success or failure of any collaborative system is recognized through the interaction of real users with the system, and additional time is required to be determine this. We hope this system will contribute to creation of a more publicly accessible, easily updatable, and reliable protein knowledge base. The experiment vindicated our User Centered Approach. The one major issue has been corrected, and in general we demonstrated the usefulness of our concept. All the groups that participated in the experiment were able to Create/Update DAS annotations from a published paper, so we consider this to demonstrate that our system is effective, usable and will provide the appropriate environment for the creation and development of a protein annotation community.

## Conclusions

We developed a system for annotating positional features on a protein sequence in a collaborative environment where the consumers of the information have the option to become authors of new annotations or to edit existing ones. From the usability experiment we learnt that DAS Writeback provides an appropriate environment for the creation, editing and deletion of protein annotations. Such a system can contribute to the curation of automatic annotation as a community process and also provides a quick way to publish manual annotations while these are awaiting annotation in a curated database. The advantage of DAS Writeback over wiki-based tools is that it enables structured, fine-grained positional annotation of sequences using existing ontologies, rather than free text, thus ensuring addition of annotation in a format compatible with the public databases.

The DAS Writeback server facilitates the collaborative annotation of biological sequences, particularly proteins, within the DAS environment. An important concept for our work is the notion of community based annotation within the biomedical domain; shifting the annotation from centralised practices to highly distributed schemas for which the participation of the community adds value to the data and improves its quality. The server was tested for performance and was found to support several concurrent users. The client was tested for usability and was found to facilitate the annotation process well.

An important milestone in the future is to provide an implementation of the same technology for other types of genetic material. For example, writeback for DNA information or for experimental information such as microarrays. We propose that future developments could include the implementation of filtering by dynamic trust rankings based on both features and users, this may achieve a higher level of confidence in the information of the writeback system.

## Availability and requirements

Project and documentation:

• Project name: writeback

• Project home: http://code.google.com/p/writeback/

• Programming language: Java+Javascript

• License: Apache 2.0

• Any restrictions to use by non-academics: None

Server:

• Project name: MyDAS (As a data source implementation)

• Project home page: http://code.google.com/p/mydas/

• Operating system(s): Platform independent

• Programming language: Java

• Other requirements: Java 1.5 or higher, Tomcat 5.0 or other servlet server.

• License: Apache 2.0

• Any restrictions to use by non-academics: None

Client:

• Project name: Dasty3 (As a plug-in)

• Project home page: http://www.ebi.ac.uk/dasty/

• Project sources: http://dasty.googlecode.com/

• Operating system(s): Platform independent

• Programming language: JavaScript-HTML

• Other requirements: JavaScript enabled in the web browser; Firefox 3.5+ is recommended

• License: Apache 2.0

• Any restrictions to use by non-academics: None

## Authors' contributions

Critical revision of the manuscript for important intellectual input: RJ, AG, HH, NM and EB. Technical and material support: HH, NM and EB. Study supervision: HH, NM and EB. Study concept: GS, RJ and AG. Architectural design: GS and EB. Software development: GS. Drafting of the manuscript: GS. Design of the usability experiment: GS, NM and EB. All authors read and approved the final manuscript.

## Supplementary Material

Additional file 1 **DAS GFF Example**. Example of a file that follows the DAS GFF format and can be used as input for the writeback server. Click here for file

Additional file 2 **Usability report**. This is list of the findings of the usability experiment for the writeback extension on Dasty. Click here for file
