# Supplementary material for: DAS Writeback: A Collaborative Annotation System
Source: BMC Bioinformatics. 2011 May 10;12:143. doi: 10.1186/1471-2105-12-143 (PMC3115852; doi:10.1186/1471-2105-12-143)
Supplement: Additional file 2 — Usability report. This is list of the findings of the usability experiment for the writeback extension on Dasty. [file 1471-2105-12-143-S2.PDF]

Usability Report

This is list of the finding of the usability experiment for the writeback extension on Dasty:

Disasters:

None

Major Problems:

- The graphic does not draw the first annotation created for a protein. The query had to be submitted again and it worked well from then onwards (4 Groups).

Minor Problems:

- The popup window to create a user should disappear automatically after a user is created. When it is still open, it creates the sensation that the user is not created (4 Groups).
- The meaning of the feature fields is not clear (4 Groups).
- The first try to get the writeback options of a feature was using right click instead the left click, that is the way it currently works (4 Groups).
- The message to inform you that a user was created is in the form of a warning, generating the impression that an error has occurred (4 Groups).
- Type ID causes problems to the users when the type is out of the ontology because they do not have an ontology Id to fillin the field, but the field is still mandatory (3 Groups).

Positive Findings:

- All the groups figured out how to add, create and delete features.
- The distinction between the original feature and the new version in the *Render as new tracks* visualization mode was intuitive for all the users.

Bugs:

- The automatic login after user creation was not working. The login panel behaved as if the user was logged in, but all the writeback functionalities were still locked (4 Groups).
- In the history of a feature, the current one should not be displayed (1 Group).

Bugs:

- Add tool tips for the fields in the forms to create and edit a feature.
- Add a function to copy or duplicate an existing annotation in order to re-use most of the values of the fields and just edit the ones that change.
- Change the name of the link to remove a deletion to Restore
- Allow the use of the Enter button to submit the data for a created/edited feature.
- Allow the use of the keyboard to navigate the component to display suggestions based on the ontology terms.
